# Triple A (Allgrove) syndrome: an unusual association with syringomyelia

**DOI:** 10.1186/1824-7288-39-39

**Published:** 2013-06-24

**Authors:** Carla Bizzarri, Danila Benevento, Cesare Terzi, Angela Huebner, Marco Cappa

**Affiliations:** 1 Endocrinology Unit, Bambino Gesú Children’s Hospital-IRCCS, Rome, Italy; 2 Department of Clinical and Experimental Medicine, University of Parma, Parma, Italy; 3 Children’s Hospital, Technical University, Dresden, Germany

**Keywords:** Allgrove syndrome, Achalasia, Alacrimia, Adrenal failure, Syringomyelia

## Abstract

Triple A (Allgrove) syndrome was first described by Allgrove in 1978 in two pairs of siblings. Since then, about 100 cases have been reported, all of them displaying an autosomal recessive pattern of inheritance. Clinical picture is characterized by achalasia, alacrimia and ACTH-resistant adrenal failure. A progressive neurological syndrome including central, peripheral and autonomic nervous system impairment, and mild mental retardation is often associated. The triple A syndrome gene, designated AAAS, is localized on chromosome 12q13. It consists of 16 exons, encoding for a 546 aminoacid protein called ALADIN (*Al*acrimia-*A*chalasia-a*D*renal *I*nsufficiency *N*eurologic disorder).

We report on a 13 year-old boy presenting with Addison’s disease, dysphagia, muscle weakness, excessive fatigue and recent onset gait ataxia. The analysis of the AAAS gene revealed a homozygous missense mutation in exon 12. It was a T > G transversion at nucleotide position 1224, resulting in a change of leucine at amino acid position 381 into arginine (Leu381Arg or L381R). Brain appearance was found normal at magnetic resonance imaging (MRI) and functional spectroscopy analysis showed normal levels of the main metabolites. Spine MRI showed a cystic cavity within the spinal cord (syringomyelia), localized between the sixth cervical vertebra and the first thoracic vertebra. Cerebellar tonsils descended 7 mm caudal to foramen magnum, consistently with a mild type 1 Chiari malformation. Mild posterior inter-vertebral disk protrusions were evident between T9 and T10 and between L4 and L5.

To our knowledge, this is the first report describing type 1 Chiari malformation and multiple spinal cord abnormalities in a patient with Allgrove syndrome.

## Background

The triple A (Allgrove) syndrome was first described in two pairs of siblings by in 1978 [1]. Since then, a number of families have been reported, all of them displaying an autosomal recessive pattern of inheritance [Online Mendelian Inheritance in Man (OMIM) database accession number: 231550].

In 1996, Weber et al. localized [2] the disease gene on chromosome 12q13. The triple A gene, designated AAAS, was cloned in 2000 by Tullio-Pelet et al. [3] and Handschug et al. [4]. The AAAS gene consists of 16 exons, encoding for a 546 amino acid protein called ALADIN (*Al*acrimia-*A*chalasia-a*D*renal *I*nsufficiency *N*eurologic disorder). The gene expression is ubiquitous, but notably high in the adrenal gland, gastrointestinal tract and brain. ALADIN protein belongs to the WD-repeat family of regulatory proteins, with functions ranging from trans membrane signaling and transcription, to cell division and intracellular trafficking [4,5]. The precise function of ALADIN is unknown, it is part of the nuclear pore complex. Missense, nonsense and splicing mutations in AAAS gene cause the protein to mislocalize to the cytoplasm [6]. Cells from subjects with Allgrove syndrome do not show morphologic abnormalities in the nuclei, nuclear envelope or nuclear pore complexes, suggesting that mutations in AAAS gene result in functional, rather than structural, abnormalities in the nuclear pore complex [7]. Frameshift, stop codon and functionally significant mutations lead to a more severe phenotype, probably occurring by a loss of function effect on the protein [8-11]. Mutations of the AAAS gene cannot be identified in all clinically diagnosed AAAS patients [4,7,10,12]. This finding raises the possibility of mutations in regulatory or deeper intronic sequences, or genetic heterogeneity.

The clinical picture is characterized by achalasia, alacrimia and ACTH-resistant adrenal failure. A progressive neurological syndrome with central, peripheral and autonomic nervous system impairment, often associated with mild mental retardation, has been described [8-11].

### Case report

**Figure 1:**
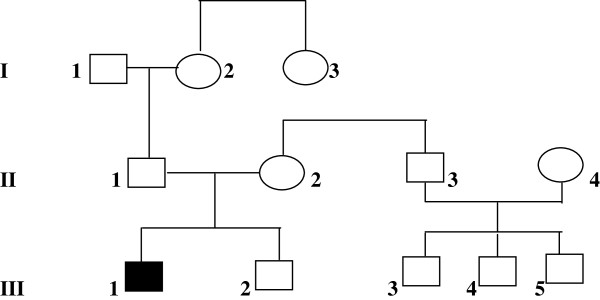
**Family pedigree. I:3**, thoracic syringomyelia with spastic diplegia at the age of 60 years, **II:4**, heterozygous adrenoleukodystrophy, spastic paraparesis, **III:3**, childhood cerebral adrenoleukodystrophy, **III:1**, the proband.

A 13 year-old boy was admitted for Addison’s disease, dysphagia, muscle weakness, excessive fatigue and recent onset gait ataxia. He was born by natural delivery, after an uneventful 40 week gestation. Birth weight was 3.3 kg, birth length 50 cm, Apgar score was 9/9. Parents were apparently healthy Caucasians of Italian ancestry, coming from the same small village of Puglia region - southern Italy. Parental consanguinity was not recognized. The younger brother of the proband was apparently healthy. As shown in Figure 1, the first son of the maternal uncle of the proband was affected by X-linked adrenoleukodystrophy (ALD). One sister of the paternal grandmother was affected by progressive spastic paresis due to a thoracic syringomyelia diagnosed at the age of 60 years.

Bilateral cryptorchidism and inguinal hernia associated with hypospadia were noted at birth, surgically repaired at the age of 6 years. At the age of 2 years, recurrent vomiting, failure to thrive and progressive skin pigmentation became evident. Gastro-esophageal reflux was diagnosed and treatment with ranitidine and domperidone was started. At the age of 4 years, he presented recurrent episodes of hypotonia, hypoglycemia and hypothermia. Addison’s disease was diagnosed and the patient was started on hydrocortisone replacement therapy.

Early developmental milestones appeared normal, with autonomous sitting at 7 months, initial speech at 12 months, walking at 14 months. A global developmental delay became evident afterwards. Psycho-educational assessment, performed when he was 9 year-old, indicated mild mental retardation.

He had a history of deficiency in tear production, dating back to early infancy, his mother described that he had always “cried without tears”.

At the age of 13 years (our first observation), height was 160 cm (0.42 SDS), weight was 71 kg (3.55 SDS), BMI: 27.73 (2.04 SDS), pubertal stage II according to Tanner, with testicular volume 6 ml bilaterally. Severe hyper-pigmentation and hyper-keratosis of elbows and knuckles were evident. Facial appearance was characterized by malar hypoplasia and prognathism, with dysartria and nasal speech. Permanent teeth were normal. Eye movements were saccadic with horizontal gaze nystagmus. Pupils were bilaterally dilated, with poor response to direct light and accommodation. Fundoscopy revealed mild bilateral optic atrophy. Distal muscle weakness and wasting were evident in the arms, more marked distally (hypothenar prominence wasting). A milder pyramidal weakness, without relevant muscle wasting was evident in the legs. He showed unsteady walking on his toes. He was unable to tandem walk and stand on either foot for more than 2 to 3 seconds. Cerebellar examination was otherwise normal. Deep tendon reflexes were hyperactive and extensor planter response was present bilaterally. Light touch, pain and temperature sensation was apparently intact.

At admission in our hospital, plasma ACTH levels were markedly elevated (1494 pg/ml – normal range: 5–46 pg/ml). Serum electrolyte levels were normal, as were serum aldosterone levels (184 pg/ml – normal values: 75–455 pg/ml) and plasma renin levels (16 pg/ml – normal values: 5–90 pg/ml). Isolated glucocorticoid deficiency, with normal mineralcorticoid balance, was confirmed and hydrocortisone therapy continued. Although X-linked ALD could be reasonably excluded from the observation of the family pedigree (Figure 1), plasma levels of very long chain fatty acids (VLCFA) were analyzed and were found normal (C26:0: 0.9 micromol/l – C26:0/C22:0: 0.008 – C24:0/C22:0: 0,77). Bone mineral density, analyzed by Dual Energy X-ray Absorptiometry (DEXA) of the lumbar spine was found normal (Z score: -0.6). Body composition analysis by DEXA showed an apparently normal proportion of lean (muscle) mass of the limbs, with increased central and peripheral fat mass.

Nerve conduction studies showed reduced sensory and motor conduction velocities in the upper and lower extremities, reduced motor responses and chronic denervation changes, consistently with a widespread peripheral sensory-motor neuropathy.

Visual evoked potentials showed marked bilateral delay and reduced amplitude. Brain stem auditory evoked potentials were normal. Somatosensory evoked potentials from the lower limbs showed mild bilateral delay and reduced amplitude.

**Figure 2:**
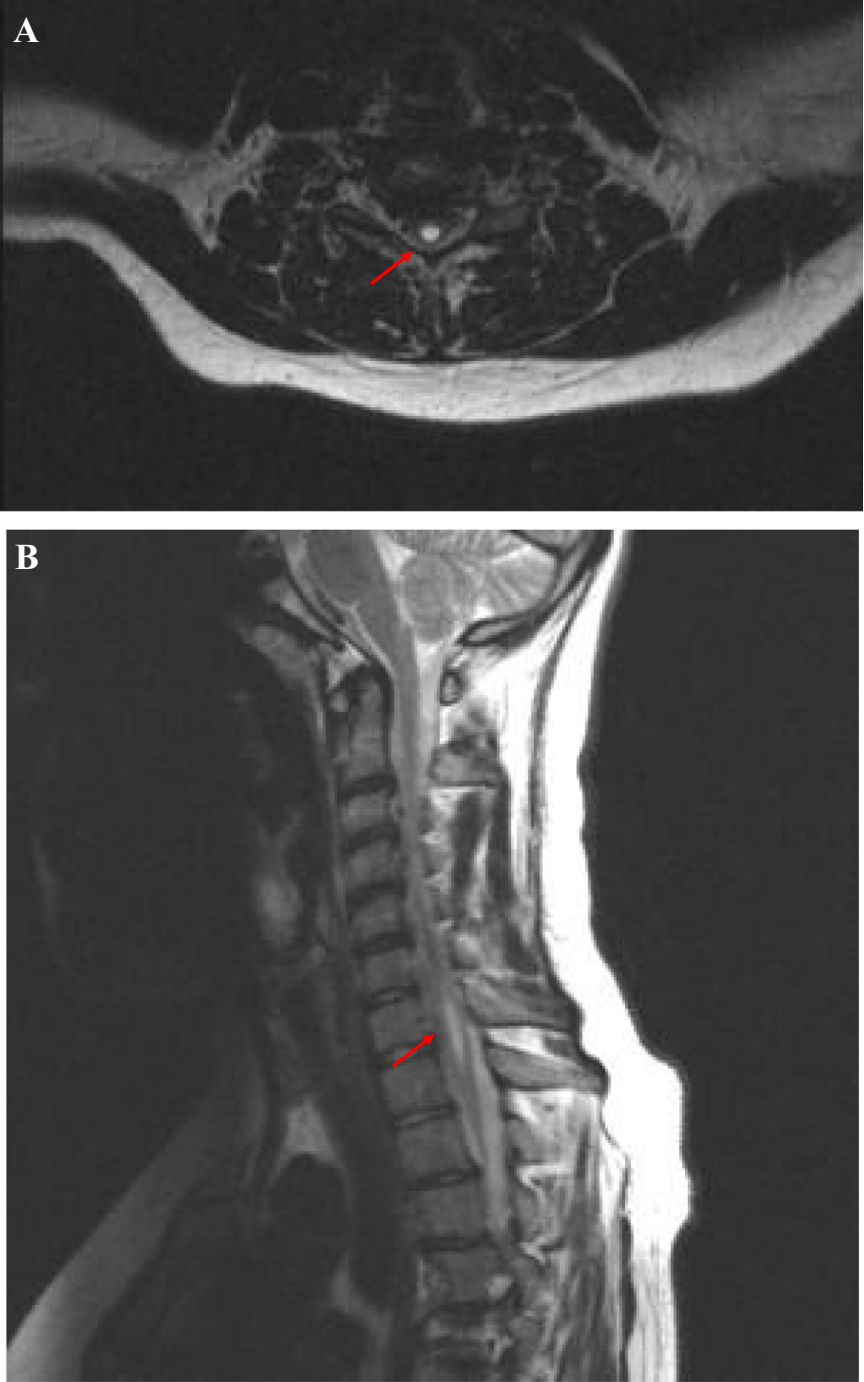
**MRI of the spine. A**: axial T2-weighed MRI of the cervical spine showing syrinx formation at the level of 6th cervical vertebra (arrow). **B**: sagittal T2-weghed MRI of the spine demonstrating type 1 Chiari malformation and associated cervico-dorsal (C6-T1) syringomyelia.

Brain magnetic resonance imaging (MRI) was found normal. Functional spectroscopy study showed normal levels of the main metabolites. Spine MRI (Figures 2A and 2B) showed a cystic cavity within the spinal cord (syringomyelia), localized between the sixth cervical vertebra (C6) and the first thoracic vertebra (T1). Maximal diameters of the cavity were mm 30 longitudinally, mm 5 front-back, mm 6 laterally. In addition, cerebellar tonsils descended 7 mm caudal to foramen magnum, consistently with a mild type 1 Chiari malformation. Mild posterior inter-vertebral disk protrusions were evident between T9 and T10 and between L4 and L5.

Our patient had manifested severe and progressive dysphagia, starting from the age of 11 years. At admission in our Hospital (aged 13) he could not swallow food without drinking. Barium swallow showed dilated esophagus and absent peristalsis. Esophageal manometry showed elevated lower esophageal sphincter (LES) pressure (54 mmHg), poor relaxation to swallow and absent body peristalsis, consistent with achalasia. He underwent Heller’s myotomy of the cardias, with good clinical results.

The genomic DNA was isolated from whole blood by proteinase K digestion and phenol/chloroform extraction. The methods of DNA amplification and sequence analysis of the AAAS gene have been previously reported in detail [7]. The analysis of the AAAS gene revealed a homozygous missense mutation in exon 12. It is a T > G transversion at nucleotide position 1224 resulting in a change of leucine at amino acid position 381 into arginine (Leu381Arg or L381R). Both parents were found heterozygous for the same mutation. No further mutations were found in the different exons or flanking intronic regions.

## Discussion

Since its first description in 1978, about 100 patients with the clinical triad of alacrimia, achalasia and adrenal insufficiency have been described. As previously reported, alacrimia was the first sign to became evident in our patient. Probably, it was already present at birth (he had never cried with tears). Recurrent vomiting, poor appetite and failure to thrive had been described by the age of 2 years, misdiagnosed as gastro-esofageal reflux. These symptoms might be related to achalasia, but recurrent vomiting associated with dehydration and failure to thrive could also represent early signs of adrenal insufficiency. In triple A syndrome, ACTH levels are usually extremely high, due to the peripheral ACTH resistance, and severe and progressive hyper-pigmentation is common. Probably, adrenal insufficiency really started in our patient by the age of 2 years, when hyper-pigmentation and gastrointestinal symptoms were first noted.

AAAS patients commonly show associated neurological abnormalities. Grant et al. [8] reported two brothers with polyneuropathy (sensory, motor and autonomic components), parkinsonian features and dementia. Cerebrospinal fluid chemistries and positron emission tomography showed evidence of abnormal dopaminergic system. Goizet et al. [9] presented a case of triple A syndrome in association with bulbo-spinal amyotrophy. Houlden et al. [10] described severe, selective ulnar nerve involvement in a patient with peripheral motor neuropathy and amyotrophy.

In our patient, the involvement of central and peripheral nervous system became evident relatively early (at the age of 13), with gait ataxia. Reduced sensory and motor conduction velocities in the upper and lower extremities, reduced motor responses and chronic denervation changes were consistent with a widespread peripheral sensory neuropathy, with both axonal and demyelinating characteristics.

Palka et al. [11] recently reported on two unrelated Italian patients showing axonal sensory motor polyneuropathy, more evident at the lower limbs. Both patients showed malar hypoplasia and prognathism, that the authors considered as probably related to the edentulism. Interestingly, our patient showed malar hypoplasia and prognathism, but presented normally erupted permanent teeth, suggesting that the facial dysmorphic features are not secondary to the teeth abnormalities, but may represent primary characteristics of the syndrome.

To our knowledge this is the first report describing Chiari 1 malformation and associated syringomyelia in a patient with Allgrove syndrome. Brooks et al. [12] described pituitary hypoplasia at MRI in one of their patients with adrenal insufficiency. Kimber et al. [13] normal MRI findings in all the analyzed patients, except for mild cerebellar atrophy in one case, and non-specific ischemic changes in the cerebral white matter in one other.

We cannot state that Chiari 1 malformation and syringomyelia are absolutely related to the AAA syndrome. Familial cases of Chiari 1 malformation, with associated syringomyelia in 30% to 85% of subjects, have been reported [14-16]. They probably represent an abnormal developmental sequence. A small posterior cranial fossa is the primary malformation, causing an anatomical predisposition to protrusion of cerebellar tonsils below the foramen magnum. Severe cases occur early in life, or during intrauterine development. Milder cases become symptomatic only after traumatic injuries or other environmental triggers [14]. Chiari 1 malformation should be the substrate for syringomyelia because of abnormal hydrodynamics of the cerebrospinal fluid in the area surrounding the foramen magnum. Progressive enlargement of the syringomyelia could impinge on the anterior horn cells and corticospinal tracts, resulting in muscle wasting of the hands, absent deep tendon reflexes in the upper extremities, and upper motor neuron signs in the lower extremities. Interruption of the anterior white commissure at the level of the cervical cord may disrupt the lateral spino-thalamic tracts, causing an asymmetric loss of pain and temperature sensation in the upper extremities. Scoliosis and multiple vertebral disk protrusions are commonly associated.

The family pedigree of our patient showed that one great-aunt was affected by thoracic syrinx, with progressive neurological symptoms starting in adult age. Our proband showed an inherited homozygous AAAs gene mutation and both parents were born in the same small village, making plausible an old estabilished, even if unrecognized, parental consanguinity. Familial Chiari 1 malformation with syringomyelia, may represent a randomly associated autosomal recessive condition [15,16], or a newly identified neurological condition related to the Allgrove syndrome itself. Conversely, X-linked adrenoleukodystrophy in the first cousin seems to be a random association, considering the different pattern of inheritance.

We could not discriminate the damage due to syringomyelia, from the specific neurological abnormalities related to AAA syndrome itself. Dysphagia and progressive dysartria could be related either to Chiari 1 malformation with associated syringomyelia or to achalasia.

A regular follow up, with repeated neurological examinations, neurophysiological studies and spinal cord MRI, will be able to clarify the influence of an eventual enlargement of the spinal cavity on the progression of the sensory-motor deficits.

In conclusion, the analysis of a large series of cases will be necessary to define whether Chiari 1 malformation and syringomyelia are anatomical abnormalities primary related to Allgrove syndrome. We propose that all patients with Allgrove syndrome undergo brain and spine MRI, even in the absence of clinical signs of neurological involvement.

### Consent

Written informed consent was obtained from the patient and his parents for publication of this Case report and accompanying images.

A copy of the written consent is available for review by the Editor-in-Chief of this journal.

## Competing interests

The authors declare that they have no competing interests.

## Authors’ contributions

CB conceived the study, participated in its design and coordination and drafted the manuscript. DB and CT carried out the clinical assessment of the case. AH performed the molecular genetic studies. MC participated in the design of the study, reviewed and edited the manuscript. All authors read and approved the final manuscript.

## References

[B1] Allgrove J Clayden GS Grant DB Macaulay JC Familial glucocorticoid deficiency with achalasia of the cardia and deficient tear production Lancet 1978 1 1284 1286 78049 10.1016/s0140-6736(78)91268-0

[B2] Weber A Wienker TF Jung M Linkage of the gene for the triple A syndrome to chromosome 12q13 near the type II keratin gene cluster Hum Mol Genet 1996 5 2061 2066 10.1093/hmg/5.12.2061 8968764

[B3] Tullio-Pelet A Salomon R Hadj-Rabia S Mugnier C De Laet MH Chaouachi B Bakiri F Brottier P Cattolico L Penet C Bégeot M Naville D Nicolino M Chaussain JL Weissenbach J Munnich A Lyonnet S Mutant WD-repeat protein in triple A syndrome Nat Genet 2000 26 332 335 10.1038/81642 11062474

[B4] Handschug K Sperling S Yoon SJ Hennig S Clark AJ Huebner A Triple A syndrome is caused by mutations in AAAS, a new WD-repeat protein gene Hum Mol Genet 2001 10 283 390 10.1093/hmg/10.3.283 11159947

[B5] Neer EJ Schmidt CJ Nambudripad R Smith TF The ancient regulatory-protein family of WD-repeat proteins Nature 1994 371 297 300 10.1038/371297a0 8090199

[B6] Cronshaw JM Matunis MJ The nuclear pore complex protein ALADIN is mislocated in triple A syndrome Proc Natl Acad Sci USA 2003 100 5823 5827 10.1073/pnas.1031047100 12730363 PMC156285

[B7] Huebner A Kaindl AM Knobeloch KP Petzold H Mann P Koehler K The triple A syndrome is due to mutations in ALADIN, a novel member of the nuclear pore complex Endocr Res 2004 30 891 899 10.1081/ERC-200044138 15666842

[B8] Grant DB Dunger DB Smith I Hyland K Familial glucocorticoid deficiency with achalasia of the cardia associated with mixed neuropathy, long tract degeneration and mild dementia Eur J Pediatr 1992 151 85 89 10.1007/BF01958948 1537368

[B9] Goizet C Catargi B Tison F Tullio-Pelet A Hadj-Rabia S Pujol F Lagueny A Lyonnet S Lacombe D Progressive bulbospinal amyotrophy in triple A syndrome with AAAS gene mutation Neurology 2002 58 962 965 10.1212/WNL.58.6.962 11914417

[B10] Houlden H Smith S De Carvalho M Blake J Mathias C Wood NW Reilly MM Clinical and genetic characterization of families with triple A (Allgrove) syndrome Brain 2002 125 2681 2690 10.1093/brain/awf270 12429595

[B11] Palka C Giuliani R Brancati F Mohn A Di Muzio A Calabrese O Huebner A De Grandis D Chiarelli F Ferlini A Stuppia L Two Italian patients with novel AAAS gene mutation expand allelic and phenotypic spectrum of triple A (Allgrove) syndrome Clin Genet 2010 77 298 301 10.1111/j.1399-0004.2009.01348.x 20447142

[B12] Brooks BP Kleta R Stuart C Tuchman M Jeong A Stergiopoulos SG Bei T Bjornson B Russell L Chanoine JP Tsagarakis S Kalsner L Stratakis C Genotypic heterogeneity and clinical phenotype in triple A syndrome: a review of NIH experience 2000–2005 Clin Genet 2005 68 215 221 10.1111/j.1399-0004.2005.00482.x 16098009

[B13] Kimber J Mc Lean BN Prevett M Hammans SR Allgrove or 4 “A” syndrome: an autosomal recessive syndrome causing multisystem neurological disease J Neurol Neurosurg Psychiatry 2003 74 654 657 10.1136/jnnp.74.5.654 12700313 PMC1738415

[B14] Szewka AJ Walsh LE Boaz JC Carvalho KS Golomb MR Chiari in the family: inheritance of the Chiari 1 malformation Pediatr Neurol 2006 34 481 485 10.1016/j.pediatrneurol.2005.09.008 16765829

[B15] Yabe I KiKuchi S Tashiro K Familial syringomyelia: the first Japanese case and review of the literature Clinical Neurology and Neurosurgery 2002 105 69 71 10.1016/S0303-8467(02)00091-4 12445928

[B16] Schijman E Hystory, aantomic forms, and pathogenesis of Chiari I malformation Child Nerv System 2004 20 323 328 10.1007/s00381-003-0878-y 14762679

